# Supplementary material for: Hot spot prediction in protein-protein interactions by an ensemble system
Source: BMC Syst Biol. 2018 Dec 31;12(Suppl 9):132. doi: 10.1186/s12918-018-0665-8 (PMC6311905; doi:10.1186/s12918-018-0665-8)
Supplement: Supplementary file 2 — All relevant MATLAB code. A simple Matlab implement of our predictor. (ZIP 19,742 kb) [file 12918_2018_665_MOESM2_ESM.zip › Additional file 2/Read ASA/ASA website/dbMPIKT/1MLCB.html]

 NetSurfP - Protein Surface Accessibility and Secondary Structure Predictions

  

|  |  |  |
| --- | --- | --- |
|  |  | NetSurfP - Protein Surface Accessibility and Secondary Structure Predictions  Technical University of Denmark |

  


---

```
# For publication of results, please cite:
# A generic method for assignment of reliability scores applied to solvent accessibility predictions.
# Bent Petersen, Thomas Nordahl Petersen, Pernille Andersen, Morten Nielsen and Claus Lundegaard
# BMC Structural Biology 2009, 9:51 doi:10.1186/1472-6807-9-51
#
# Column 1: Class assignment - B for buried or E for Exposed - Threshold: 25% exposure, but not based on RSA
# Column 2: Amino acid
# Column 3: Sequence name
# Column 4: Amino acid number
# Column 5: Relative Surface Accessibility - RSA
# Column 6: Absolute Surface Accessibility
# Column 7: Z-fit score for RSA prediction
# Column 8: Probability for Alpha-Helix
# Column 9: Probability for Beta-strand
# Column 10: Probability for Coil
E Q  1MLCB                  1    0.804 143.666   1.330   0.003   0.003   0.994
B V  1MLCB                  2    0.202  31.078  -0.162   0.004   0.616   0.381
E Q  1MLCB                  3    0.428  76.405   1.326   0.001   0.900   0.099
B L  1MLCB                  4    0.148  27.135   0.691   0.002   0.816   0.182
E Q  1MLCB                  5    0.399  71.208   0.622   0.021   0.756   0.223
B E  1MLCB                  6    0.261  45.579   0.768   0.004   0.514   0.481
E S  1MLCB                  7    0.438  51.287  -0.576   0.020   0.205   0.775
E G  1MLCB                  8    0.491  38.634  -1.748   0.018   0.088   0.893
E A  1MLCB                  9    0.514  56.632  -2.143   0.018   0.047   0.935
E E  1MLCB                 10    0.496  86.721  -1.605   0.020   0.205   0.775
E V  1MLCB                 11    0.407  62.633  -0.832   0.021   0.451   0.528
E M  1MLCB                 12    0.295  58.969   0.749   0.021   0.451   0.528
E K  1MLCB                 13    0.436  89.726   1.213   0.020   0.205   0.775
E P  1MLCB                 14    0.449  63.713  -0.552   0.005   0.045   0.951
E G  1MLCB                 15    0.621  48.873  -1.978   0.005   0.045   0.951
E A  1MLCB                 16    0.419  46.119  -0.697   0.004   0.138   0.858
E S  1MLCB                 17    0.443  51.966  -0.336   0.004   0.420   0.576
B V  1MLCB                 18    0.153  23.593   0.719   0.002   0.816   0.182
E K  1MLCB                 19    0.427  87.772   0.788   0.001   0.900   0.099
B I  1MLCB                 20    0.080  14.708   0.452   0.001   0.900   0.099
B S  1MLCB                 21    0.189  22.163   0.653   0.001   0.900   0.099
B C  1MLCB                 22    0.045   6.248   0.074   0.001   0.900   0.099
B K  1MLCB                 23    0.289  59.509   0.451   0.001   0.900   0.099
B A  1MLCB                 24    0.116  12.761  -0.356   0.002   0.816   0.182
B T  1MLCB                 25    0.171  23.704  -0.703   0.004   0.514   0.481
B G  1MLCB                 26    0.245  19.258  -1.933   0.004   0.197   0.799
B Y  1MLCB                 27    0.238  50.861  -1.071   0.005   0.336   0.660
B T  1MLCB                 28    0.194  26.908  -0.495   0.004   0.514   0.481
B F  1MLCB                 29    0.098  19.628  -1.242   0.021   0.451   0.528
B S  1MLCB                 30    0.213  24.917  -1.105   0.021   0.279   0.699
B T  1MLCB                 31    0.283  39.266  -1.308   0.018   0.088   0.893
B Y  1MLCB                 32    0.110  23.464  -0.027   0.019   0.141   0.840
B W  1MLCB                 33    0.086  20.707   0.143   0.022   0.359   0.619
B I  1MLCB                 34    0.030   5.624   0.064   0.023   0.655   0.322
B E  1MLCB                 35    0.048   8.386  -0.174   0.021   0.756   0.223
B W  1MLCB                 36    0.035   8.466   0.369   0.018   0.846   0.136
B V  1MLCB                 37    0.036   5.533   0.188   0.018   0.846   0.136
B K  1MLCB                 38    0.116  23.902   0.325   0.021   0.756   0.223
B Q  1MLCB                 39    0.083  14.860   0.373   0.021   0.451   0.528
B R  1MLCB                 40    0.223  51.113   0.798   0.005   0.045   0.951
E P  1MLCB                 41    0.356  50.545   0.757   0.005   0.015   0.979
E G  1MLCB                 42    0.576  45.363  -1.671   0.018   0.019   0.964
E H  1MLCB                 43    0.385  69.995   0.749   0.018   0.019   0.964
E G  1MLCB                 44    0.441  34.722  -0.391   0.018   0.088   0.893
B L  1MLCB                 45    0.157  28.765   1.110   0.005   0.262   0.733
B E  1MLCB                 46    0.100  17.400  -0.221   0.004   0.616   0.381
B W  1MLCB                 47    0.044  10.558  -0.050   0.001   0.900   0.099
B I  1MLCB                 48    0.024   4.459   0.919   0.001   0.959   0.040
B G  1MLCB                 49    0.017   1.362   0.086   0.001   0.959   0.040
B E  1MLCB                 50    0.074  12.875  -0.439   0.001   0.959   0.040
B I  1MLCB                 51    0.020   3.774   0.964   0.001   0.959   0.040
B L  1MLCB                 52    0.154  28.142   0.506   0.002   0.816   0.182
B P  1MLCB                 53    0.218  30.877  -0.620   0.005   0.336   0.660
E G  1MLCB                 54    0.522  41.058  -1.446   0.005   0.045   0.951
E S  1MLCB                 55    0.602  70.590  -2.542   0.003   0.003   0.994
E G  1MLCB                 56    0.566  44.513  -2.265   0.018   0.019   0.964
E S  1MLCB                 57    0.455  53.326  -0.728   0.018   0.047   0.935
E T  1MLCB                 58    0.337  46.686  -0.256   0.019   0.141   0.840
E Y  1MLCB                 59    0.435  92.959  -0.052   0.020   0.205   0.775
B Y  1MLCB                 60    0.216  46.202   0.151   0.020   0.205   0.775
B N  1MLCB                 61    0.224  32.808  -0.322   0.018   0.088   0.893
E E  1MLCB                 62    0.620 108.366  -0.800   0.018   0.047   0.935
E K  1MLCB                 63    0.438  90.199   0.026   0.018   0.047   0.935
B F  1MLCB                 64    0.096  19.187  -0.226   0.019   0.141   0.840
E K  1MLCB                 65    0.405  83.391   0.701   0.004   0.197   0.799
B G  1MLCB                 66    0.192  15.110  -1.507   0.004   0.420   0.576
B K  1MLCB                 67    0.210  43.176   0.741   0.002   0.816   0.182
B A  1MLCB                 68    0.055   6.094  -0.312   0.001   0.900   0.099
B T  1MLCB                 69    0.134  18.558   0.831   0.001   0.959   0.040
B F  1MLCB                 70    0.049   9.794   0.893   0.001   0.900   0.099
B T  1MLCB                 71    0.125  17.310   0.116   0.003   0.718   0.279
B A  1MLCB                 72    0.260  28.685   0.376   0.022   0.552   0.426
B D  1MLCB                 73    0.264  38.014  -0.675   0.005   0.336   0.660
E T  1MLCB                 74    0.435  60.279  -0.126   0.018   0.088   0.893
B S  1MLCB                 75    0.264  30.882  -1.560   0.018   0.088   0.893
E S  1MLCB                 76    0.511  59.831   0.773   0.019   0.141   0.840
B N  1MLCB                 77    0.251  36.702  -0.455   0.022   0.359   0.619
B T  1MLCB                 78    0.204  28.336   0.920   0.021   0.756   0.223
B A  1MLCB                 79    0.082   9.025  -0.219   0.011   0.918   0.071
B Y  1MLCB                 80    0.173  36.991   0.231   0.011   0.918   0.071
B M  1MLCB                 81    0.051  10.225   0.595   0.011   0.918   0.071
B Q  1MLCB                 82    0.163  29.076  -0.091   0.011   0.918   0.071
B L  1MLCB                 83    0.083  15.234  -0.695   0.021   0.756   0.223
E S  1MLCB                 84    0.371  43.481  -0.025   0.021   0.451   0.528
E S  1MLCB                 85    0.388  45.497  -0.913   0.004   0.197   0.799
B L  1MLCB                 86    0.129  23.675  -0.002   0.019   0.141   0.840
E T  1MLCB                 87    0.485  67.214   1.129   0.018   0.088   0.893
E S  1MLCB                 88    0.369  43.282  -0.695   0.052   0.084   0.864
E E  1MLCB                 89    0.474  82.825  -0.659   0.053   0.043   0.903
B D  1MLCB                 90    0.179  25.808  -0.688   0.018   0.088   0.893
B S  1MLCB                 91    0.111  13.068   0.484   0.021   0.451   0.528
B A  1MLCB                 92    0.035   3.879   0.004   0.018   0.846   0.136
B V  1MLCB                 93    0.061   9.422   1.174   0.011   0.918   0.071
B Y  1MLCB                 94    0.034   7.373   0.765   0.011   0.918   0.071
B Y  1MLCB                 95    0.050  10.770   0.646   0.011   0.918   0.071
B C  1MLCB                 96    0.014   2.022   1.592   0.018   0.846   0.136
B A  1MLCB                 97    0.028   3.042   0.229   0.004   0.616   0.381
B R  1MLCB                 98    0.135  31.007   0.377   0.005   0.336   0.660
B G  1MLCB                 99    0.253  19.935  -0.463   0.004   0.138   0.858
E D  1MLCB                100    0.364  52.409  -1.126   0.018   0.047   0.935
E G  1MLCB                101    0.380  29.937  -1.861   0.018   0.019   0.964
B N  1MLCB                102    0.227  33.277  -2.077   0.018   0.088   0.893
E Y  1MLCB                103    0.305  65.243  -1.187   0.019   0.141   0.840
B G  1MLCB                104    0.270  21.233  -0.856   0.019   0.141   0.840
B Y  1MLCB                105    0.238  50.925  -0.655   0.020   0.205   0.775
B W  1MLCB                106    0.103  24.747  -1.866   0.004   0.197   0.799
B G  1MLCB                107    0.076   6.005  -3.033   0.021   0.279   0.699
B Q  1MLCB                108    0.207  37.006  -0.698   0.004   0.197   0.799
B G  1MLCB                109    0.131  10.318  -1.854   0.004   0.197   0.799
B T  1MLCB                110    0.186  25.840  -0.514   0.022   0.359   0.619
E T  1MLCB                111    0.327  45.327  -0.515   0.022   0.359   0.619
B L  1MLCB                112    0.100  18.292  -1.347   0.021   0.279   0.699
E T  1MLCB                113    0.351  48.670  -0.632   0.022   0.359   0.619
B V  1MLCB                114    0.221  33.906  -0.454   0.021   0.451   0.528
B S  1MLCB                115    0.254  29.781  -1.246   0.020   0.205   0.775
E S  1MLCB                116    0.395  46.306  -1.040   0.018   0.047   0.935
E A  1MLCB                117    0.502  55.298  -1.281   0.018   0.047   0.935
E S  1MLCB                118    0.379  44.384  -0.609   0.018   0.047   0.935
E T  1MLCB                119    0.521  72.304  -0.787   0.018   0.088   0.893
E T  1MLCB                120    0.494  68.448  -1.184   0.019   0.141   0.840
E P  1MLCB                121    0.495  70.240  -1.053   0.020   0.205   0.775
E P  1MLCB                122    0.369  52.404  -0.686   0.021   0.279   0.699
E S  1MLCB                123    0.554  64.905  -0.681   0.021   0.451   0.528
E V  1MLCB                124    0.361  55.563  -0.328   0.022   0.359   0.619
B F  1MLCB                125    0.304  60.993  -0.887   0.021   0.279   0.699
E P  1MLCB                126    0.420  59.584  -1.249   0.064   0.216   0.721
B L  1MLCB                127    0.237  43.395  -1.692   0.064   0.216   0.721
E A  1MLCB                128    0.402  44.333  -1.242   0.064   0.216   0.721
B P  1MLCB                129    0.301  42.712  -2.321   0.056   0.142   0.802
E G  1MLCB                130    0.393  30.937  -2.175   0.018   0.088   0.893
E S  1MLCB                131    0.471  55.201  -1.827   0.018   0.047   0.935
E A  1MLCB                132    0.417  45.997  -1.379   0.056   0.142   0.802
E A  1MLCB                133    0.516  56.819  -1.548   0.056   0.142   0.802
E Q  1MLCB                134    0.484  86.496  -1.384   0.019   0.141   0.840
E T  1MLCB                135    0.599  83.095  -1.372   0.018   0.088   0.893
E N  1MLCB                136    0.471  68.954  -1.678   0.018   0.088   0.893
E S  1MLCB                137    0.511  59.901  -1.158   0.004   0.197   0.799
E M  1MLCB                138    0.379  75.838  -0.544   0.005   0.336   0.660
B V  1MLCB                139    0.134  20.611  -0.288   0.004   0.616   0.381
B T  1MLCB                140    0.200  27.712  -0.336   0.002   0.816   0.182
B L  1MLCB                141    0.031   5.731  -0.057   0.001   0.900   0.099
B G  1MLCB                142    0.061   4.816  -0.375   0.002   0.816   0.182
B C  1MLCB                143    0.020   2.752   0.896   0.002   0.816   0.182
B L  1MLCB                144    0.148  27.044   0.470   0.001   0.900   0.099
B V  1MLCB                145    0.029   4.396  -0.756   0.001   0.900   0.099
B K  1MLCB                146    0.206  42.436   0.103   0.004   0.514   0.481
E G  1MLCB                147    0.279  21.989  -2.201   0.005   0.262   0.733
B Y  1MLCB                148    0.188  40.133  -1.138   0.005   0.336   0.660
B F  1MLCB                149    0.255  51.158  -0.597   0.004   0.420   0.576
B P  1MLCB                150    0.194  27.500  -1.216   0.004   0.420   0.576
E E  1MLCB                151    0.438  76.466  -0.903   0.005   0.262   0.733
E P  1MLCB                152    0.418  59.371  -1.405   0.004   0.197   0.799
B V  1MLCB                153    0.135  20.734   0.329   0.004   0.616   0.381
B T  1MLCB                154    0.180  24.994  -0.704   0.002   0.816   0.182
B V  1MLCB                155    0.043   6.655  -0.176   0.011   0.918   0.071
B T  1MLCB                156    0.133  18.447  -0.347   0.011   0.918   0.071
B W  1MLCB                157    0.051  12.265   0.417   0.018   0.846   0.136
B N  1MLCB                158    0.195  28.577   0.333   0.018   0.846   0.136
E S  1MLCB                159    0.375  43.962  -0.287   0.004   0.616   0.381
B G  1MLCB                160    0.272  21.438  -0.270   0.004   0.197   0.799
E S  1MLCB                161    0.504  59.127   0.080   0.005   0.015   0.979
E L  1MLCB                162    0.469  85.911  -0.754   0.005   0.015   0.979
E S  1MLCB                163    0.641  75.090  -2.234   0.005   0.045   0.951
E S  1MLCB                164    0.472  55.318   0.235   0.005   0.045   0.951
E G  1MLCB                165    0.426  33.542  -0.756   0.004   0.138   0.858
B V  1MLCB                166    0.155  23.808   0.002   0.004   0.514   0.481
B H  1MLCB                167    0.286  51.969  -0.178   0.001   0.900   0.099
B T  1MLCB                168    0.234  32.497  -0.508   0.001   0.900   0.099
B F  1MLCB                169    0.133  26.653   1.259   0.001   0.900   0.099
B P  1MLCB                170    0.081  11.551  -1.359   0.003   0.718   0.279
B A  1MLCB                171    0.211  23.285  -1.164   0.001   0.900   0.099
B V  1MLCB                172    0.102  15.739  -1.084   0.001   0.900   0.099
B L  1MLCB                173    0.271  49.620  -0.012   0.003   0.718   0.279
E Q  1MLCB                174    0.535  95.551  -1.014   0.005   0.336   0.660
E S  1MLCB                175    0.725  84.935  -1.777   0.005   0.015   0.979
E D  1MLCB                176    0.599  86.330  -2.874   0.005   0.015   0.979
E L  1MLCB                177    0.421  77.158  -0.146   0.005   0.336   0.660
B Y  1MLCB                178    0.235  50.155  -1.027   0.004   0.616   0.381
B T  1MLCB                179    0.207  28.655  -0.858   0.002   0.816   0.182
B L  1MLCB                180    0.149  27.264  -0.882   0.001   0.900   0.099
B S  1MLCB                181    0.258  30.214  -0.607   0.001   0.900   0.099
B S  1MLCB                182    0.045   5.274  -0.803   0.001   0.900   0.099
B S  1MLCB                183    0.209  24.495   0.363   0.001   0.959   0.040
B V  1MLCB                184    0.046   7.039   0.226   0.001   0.959   0.040
B T  1MLCB                185    0.221  30.653   0.605   0.001   0.959   0.040
B V  1MLCB                186    0.058   8.838   0.800   0.001   0.900   0.099
E P  1MLCB                187    0.314  44.628   0.719   0.004   0.616   0.381
E S  1MLCB                188    0.356  41.723  -0.422   0.021   0.279   0.699
E S  1MLCB                189    0.590  69.148  -1.344   0.005   0.262   0.733
E P  1MLCB                190    0.588  83.494   0.388   0.004   0.197   0.799
B R  1MLCB                191    0.198  45.296  -1.007   0.004   0.138   0.858
E P  1MLCB                192    0.714 101.288   1.069   0.005   0.045   0.951
E S  1MLCB                193    0.692  81.056   0.680   0.005   0.045   0.951
E E  1MLCB                194    0.446  77.846   0.899   0.004   0.514   0.481
E T  1MLCB                195    0.399  55.300   1.486   0.001   0.900   0.099
B V  1MLCB                196    0.070  10.820   0.886   0.001   0.959   0.040
E T  1MLCB                197    0.306  42.428   1.732   0.001   0.959   0.040
B C  1MLCB                198    0.024   3.426   0.396   0.001   0.959   0.040
E N  1MLCB                199    0.344  50.435   0.982   0.001   0.959   0.040
B V  1MLCB                200    0.071  10.913  -0.725   0.001   0.900   0.099
E A  1MLCB                201    0.523  57.646   0.614   0.001   0.900   0.099
B H  1MLCB                202    0.187  34.088   0.158   0.004   0.514   0.481
E P  1MLCB                203    0.620  87.964  -0.064   0.005   0.045   0.951
E A  1MLCB                204    0.696  76.732  -1.345   0.005   0.015   0.979
E S  1MLCB                205    0.550  64.507  -0.539   0.004   0.085   0.910
E S  1MLCB                206    0.575  67.378  -0.124   0.004   0.138   0.858
E T  1MLCB                207    0.435  60.307   0.572   0.005   0.336   0.660
E K  1MLCB                208    0.555 114.081   1.556   0.003   0.718   0.279
B V  1MLCB                209    0.096  14.740   0.049   0.002   0.816   0.182
E D  1MLCB                210    0.639  92.022   0.297   0.001   0.900   0.099
E K  1MLCB                211    0.269  55.251   1.242   0.021   0.756   0.223
E K  1MLCB                212    0.588 121.054   0.886   0.003   0.718   0.279
E I  1MLCB                213    0.251  46.361   0.304   0.004   0.420   0.576
E V  1MLCB                214    0.475  72.977   0.123   0.004   0.138   0.858
E P  1MLCB                215    0.546  77.520  -0.855   0.004   0.085   0.910
E R  1MLCB                216    0.660 151.163  -0.325   0.018   0.047   0.935
E D  1MLCB                217    0.578  83.232  -0.909   0.018   0.019   0.964
E C  1MLCB                218    0.639  89.786  -2.011   0.000   0.000   0.999

Explain the output.  Go back.
```

---
